# Effects of nature on restorative and cognitive benefits in indoor environment

**DOI:** 10.1038/s41598-023-40408-x

**Published:** 2023-08-14

**Authors:** Jee Heon Rhee, Brian Schermer, Gisung Han, So Yeon Park, Kyung Hoon Lee

**Affiliations:** 1 School of Architecture and Urban Planning, University of Wisconsin-Milwaukee, Milwaukee, WI USA; 2 Institute of Engineering Research, Korea University, Seoul, Korea; 3 Department of Urbanism, Faculty of Architecture and the Built Environment, Delft University of Technology (TU Delft), Delft, The Netherlands; 4 Department of Architecture, Korea University, Seoul, Korea

**Keywords:** Psychology, Human behaviour, Attention, Working memory

## Abstract

This study investigates the effects of natural exposure in an indoor environment on restorative quality and cognitive ability. Thirty participants were shown nature at three different indoor sites: baseline, indoor (some vegetation), and semi-indoor (a large amount of vegetation and view to sky) for five minutes. After viewing, they completed an assessment of restoration and a cognitive task, and their electroencephalography (EEG) was recorded. Compared to the baseline, the sites with nature resulted in restorative (higher perceived restoration scores) and cognitive (higher working memory performance and lower delta-to-theta ratio (DTR), delta-to-alpha ratio (DAR), theta-to-beta ratio (TBR), and alpha-to-beta ratio (ABR) responses) benefits. These findings further our understanding of the effects of exposure to nature on restorative and cognitive benefits in an indoor environment, and help to build guidance for future research on the effects of nature indoors and designing restorative- and cognitive-enhancing indoor spaces.

## Introduction

Nature has a positive effect on people. Previous studies have demonstrated its restorative and cognitive benefits^1–3^, based on theories regarding the relationship between people and nature, compared to the built environment^4^. Outdoor field studies have shown that walking in a natural environment enhances attention restoration^5^ and increases cognitive task scores^6^. Images and videos of natural and built environments have been used to evaluate the positive effects of natural environments on restoration and cognition^7,8^.

The restorative and cognitive benefits of natural environments can be well explained and supported by Attention Restoration Theory (ART)^9,10^. ART^4,11^, which suggests that natural environments enable people to restore depleted attention and improve cognitive functioning. ART claims that directed attention is required to maintain cognitive clarity to effectively carry out daily routines. Unfortunately, the directed attention capacity is limited and often exhausted, owing to its extensive use in modern society. Its extensive use causes mental fatigue and the depletion of cognitive resources^12^. ART suggests that exposure to natural environments can aid in restoring directed attention^6^.

According to ART, four components of environmental experiences, namely “Fascination,” “Being away,” “Extent,” and “Compatibility,” mitigate mental fatigue and restore attention^4,11^. Fascination refers to effortless attention evoked by the environment, and being away refers to psychological and geographical distancing from routines such as daily obligations. Extent comprises two sub-components, scope and coherence; scope relates to a physical scale of the environment and the quantity of engagement that individuals are exposed to, and coherence relates to how elements in an environment connect with their structure and organization as a whole. Compatibility refers to the match or congruence between a person’s intentions and environmental assistance. Empirical studies on ART have confirmed that natural environments offer these components to a higher degree than built environments^13^. Based on these theoretical studies, various versions of the Perceived Restorativeness Scale (PRS) have been developed and used to evaluate the restorative quality of environments^14,15^.

A meta-analysis of the effect of natural environments on cognitive ability confirmed that exposure to nature affects working memory most significantly among the eight cognitive domains^16^. Relatively demanding attentional tasks have been suggested as a method to quantify the effects of natural environment on cognitive ability^17^. According to ART, direct attention requires relatively more cognitive resources than involuntary attention^18^. Thus, demanding attentional tasks, such as backward digit span, have been used as one of the well-known tasks to measure working memory^17^.

Although there is much more emphasis on outdoor nature, several studies have investigated the beneficial effects of indoor nature, including its restorative and cognitive benefits^17,19,20^. The effects of indoor nature on cognitive benefits have been further studied^21,22^, but the body of existing knowledge is insufficient and the existing results are inconsistent^19,23^. Elementary school students showed higher attention task scores in a classroom with an indoor green wall^24^, while university students showed higher reading span task results in office spaces with plants^25^. In contrast, Van den Bogerd, et al.^26^ and Evensen, et al.^27^ did not find significant differences in cognitive abilities with and without indoor nature in classroom and office spaces, respectively. The restorative benefits of indoor nature have not been studied enough. Han^28^ used the Restorative Components Scale (RCS) and the Restoration Scale (RS) to measure the restorative quality of plants, but did not find significant differences in the classroom with and without plants. In short, both the restorative and cognitive effects of indoor nature are not as clear as those of outdoor natural environments.

Electroencephalography (EEG) responses have been adopted as a measure of the effects of nature. Grassini, et al.^29^ found that event-related potentials in EEG responses were related to lower attentional and cognitive load when images of outdoor nature scenery were provided to the participants. Chen, et al.^30^ used alpha-theta synchronization and oscillations in association with attention restoration, and observed stronger alpha-theta synchronization and oscillations in an outdoor natural environment than in an outdoor urban environment. Nevertheless, only a few studies have employed EEG to explore the effects of exposure to nature on cognitive abilities, let alone the effects of exposure to nature in indoor environments.

Ratios between frequency band powers based on EEG responses have been adopted as an indication of cognitive ability. A higher theta-to-beta ratio (TBR) was found to represent lower attentional capacity^31,32^. Specifically, the TBR during the resting state is negatively associated with attentional control^33,34^, attention restoration^34^, and cognitive processing capacity^35^. Decreased alpha-to-beta ratio (ABR) has been considered a quantitative marker of attention^36,37^ because decreased alpha and increased beta reflect states of enhanced attention^38^. The delta-to-theta (DTR) and delta-to-alpha ratios (DAR) have been investigated in relation to cognitive function, although their mechanisms have not been sufficiently explored. Recent studies have found that they are related to mental workload and cognitive stress in the frontal and occipital regions^39–41^. While these measures have been regarded as a means of representing cognitive abilities, they have not been adopted to investigate the effect of indoor nature.

The objective of this study is to examine the effects of indoor nature on real-world sites using PRS-11, backward digit span, and EEG responses such as DTR, DAR, TBR, and ABR. We hypothesize that an indoor environment with exposure to nature offers higher restorative quality and better cognitive ability than an indoor environment without nature. This study aims to clarify the restorative and cognitive benefits of indoor nature using various measurement methods in real-world settings. In addition, we aim to associate an extensive set of EEG responses with cognitive abilities in relation to ART, particularly in the context of indoor settings.

## Results

### PRS-11

Table 1 shows the mean PRS-11 scores (mean and standard deviation) and repeated-measures ANOVA results for the experimental sites (F and *p*-value). Bonferroni post hoc tests showed that the restorative quality of the Sites C and B was significantly higher in PRS-general, Fascination, Being away, and Scope than Site A. In addition, the restorative quality of Site C was higher than that of Site B in both PRS-general and Fascination. In contrast, the restorative quality of Sites C and B was significantly lower in Coherence than in Site A.

**Table 1:** Mean Number of the PRS-11 and the Backward Digit Span Scores of Experiment Sites.

	Experiment sites (N = 30)				
	Baseline site (A)	Indoor site (B)	Semi-indoor site (C)	F	*p*
PRS-11					
PRS_Gen: Mean (SD)	3.52 (1.80)^a^	6.84 (1.25)^b^	7.56 (0.94)^c^	77.858	0.000***
Fascination:Mean (SD)	3.07 (1.93)^a^	7.03 (1.33)^b^	7.98 (1.12)^c^	96.248	0.000***
Being Away: Mean (SD)	3.64 (2.54)^a^	6.97 (1.61)^b^	7.56 (1.39)^b^	34.713	0.000***
Coherence: Mean (SD)	7.03 (2.25)^a^	5.52 (1.45)^b^	4.97 (1.57)^b^	11.033	0.000***
Scope: Mean (SD)	4.00 (1.83)^a^	6.37 (1.76)^b^	6.93 (1.43)^b^	33.955	0.000***
Backward Digit Span					
Scores: Mean (SD)	7.50 (2.57)	7.70 (2.56)	8.10 (2.37)	1.012	0.370

***p < .005, Bonferroni correction: a < b < c.

### Backward digit span

Table 1 shows the results of the backward digit span scores at different sites and the repeated measures ANOVA results. Although differences in the mean score were observed among the three sites, the ANOVA results did not show a statistically significant difference among them.

### EEG

**Figure 1:**
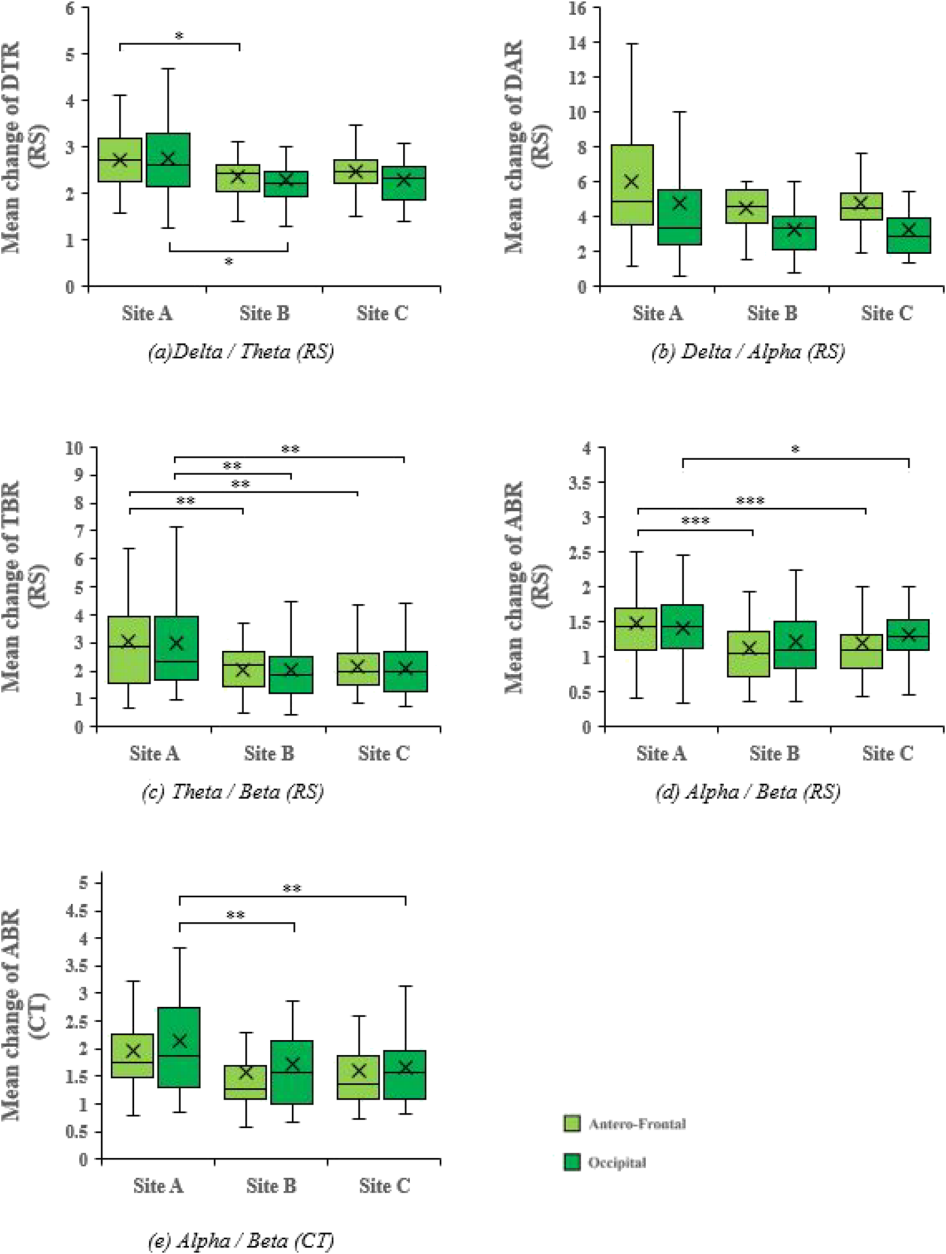
Mean changes in EEG in anterofrontal and occipital region during resting states (RS) and cognitive tasks (CT) (*p < .05, **p < .005, ***p < .001).

Figure 1 shows the results of the repeated-measures ANOVA of EEG in the anterofrontal and occipital regions during resting states and cognitive tasks. The three sites showed statistically significant differences in DTR in the anterofrontal (F [2, 58] = 3.755, *p = *0.029, ɳp^2^ = 0.115) and occipital (F [2, 58] = 5.034, *p = *0.010, ɳp^2^ = 0.148) regions. Site A was significantly higher in the anterofrontal and occipital DTRs compared to Site B, according to the Bonferroni post-hoc test. The three sites also showed a statistically significant difference in DAR in anterofrontal (F [1.402, 40.651] = 4.200, *p = *0.034, ɳp^2^ = 0.127) and occipital (F [1.582, 45.871] = 5.311, *p = *0.013, ɳp^2^ = 0.155) regions although Bonferroni post hoc test did not show significant differences. Furthermore, a significant difference among the three sites was observed in the TBR in the anterofrontal (F [1.299, 37.669] = 8.213, *p = *0.004, ɳp^2^ = 0.221) and occipital (F [1.405, 40.753] = 7.611, *p = *0.004, ɳp^2^ = 0.208) regions. Site A had significantly higher anterofrontal and occipital TBRs than Sites B and C. In addition, there was a significant difference in ABR among the three sites in the anterofrontal (F [2, 58] = 10.146, *p = *0.000, ɳp^2^ = 0.259) and occipital (F [1.602, 46.461] = 4.819, *p = *0.018, ɳp^2^ = 0.142) regions. Bonferroni post hoc test indicated that Site A showed a significantly higher anterofrontal ABR than Site B and higher anterofrontal and occipital ABRs than Site C. Finally, there was a significant difference among the three sites regarding occipital ABR during the cognitive tasks (F [2, 58] = 8.473, *p = *0.001, ɳp^2^ = 0.226). Site A was significantly higher in the occipital ABR than Sites B and C.

## Discussion

This study explored the relationships between restorative quality and cognitive ability in indoor environments with and without nature in real-world settings. Consistent with previous studies^14,15,42^, we found that the presence of nature in the indoor environment was related to increased restorative quality and affected all the PRS components. PRS-General, Fascination, Being Away, and Scope increased when exposed to indoor conditions, with Site C showing the highest PRS-General and Fascination scores, followed by Site B and Site A. Sites B and C were equivalent in Being Away and Scope scores and were higher compared to Site A. In contrast, Coherence decreased in Sites B and C compared to Site A. This result is also consistent with previous studies showing that built environments without nature had higher Coherence than natural environments^9,14^.

The results showed that Site C had a significantly higher restorative quality in PRS-General and Fascination than Site B. Sites B and C are different with varying environmental characteristics. One of the notable differences between the two sites is the different levels of vegetation and view of the sky. The higher restorative quality in Site C may be attributed to the density of nature (i.e., more vegetation and sky). This reasoning aligns with existing evidence that more vegetation^43^ and sky^44^ are positively associated with restorative benefits. Carrus, et al.^43^ found that the level of naturalness is positively associated with PRS, and Masoudinejad and Hartig^44^ found that images with higher sky ratios increased Fascination and Being away from PRS as well as restoration likelihood.

Our results, based on the backward digit span task, showed that the effect of the indoor nature on working memory was not statistically significant. In fact, the existing body of literature shows inconsistent results regarding the effects of nature on working memory. A study conducted in real and virtual environments found a 14% increase in working memory only in real environments with nature^45^. In addition, a comparison study of nature and urban walking experience showed that working memory is improved only with measurements by the automated operation span task, but the backward digit span scores did not show significant differences^46^. Moreover, the effect of indoor nature on working memory may be impacted by the exposure time to nature; in our experiment, participants viewed each site for five minutes. Previous studies with a longer exposure duration showed significantly improved backward digit span scores after a 50–55 min walk in nature compared to an urban environment^6^ and after two months with plants in the classroom^47^.

The EEG analysis indicated that the TBR and ABR of Sites B and C were significantly lower than those of Site A, except in one case (occipital ABR between Sites A and B). Previous studies^31,32^ have confirmed the association between TBR and ABR with attention. This evidence suggests that the reduced anterofrontal and occipital TBRs and ABRs in the presence of nature in our experiment may indicate increased attention. The anterofrontal and occipital DTRs and DARs were significantly different among the sites, with a trend of lower mean values toward indoor nature sites (B, C), but the post hoc tests confirmed that only the anterofrontal and occipital DTRs of Site B were significantly lower than those of Site A. Lower DTR and DAR have been reported to be associated with lower mental workloads and cognitive stress^40,41^. Thus, it can be inferred that the lower anterofrontal and occipital DTRs and DARs in natural indoor environments may result in lower mental workload and cognitive stress. These results can be reinforced by ART, as the presence of nature in the indoor environment enhances people’s attention capacity by restoring depleted attention from everyday life^4^.

In the EEG analysis, DTR, DAR, TBR, and ABR showed significant differences during resting states; however, during the cognitive task, only occipital ABR showed statistically significant results in the presence of nature (Sites B and C) compared to Site A. There were no significant differences between Sites B and C during the cognitive task. Differences in occipital ABR between the resting state and cognitive task were 0.072, 0.155, and 0.164 for Sites C, B, and A, respectively. The higher occipital ABR during the cognitive task is explained by the fact that participants are most likely to focus on the given task and experience more mental workload than during the resting state. Moreover, during the cognitive task, the effect of indoor nature was found to be statistically insignificant for most of the EEG responses, implying that perceived exposure to nature may be influenced by the level of attention.

Our findings reveal that participants perceived a higher level of restoration in Site C compared to Site B, although there were no significant differences in the EEG results between these two sites. A recent study by Rhee et al.^48^ also points out that perceived restoration and EEG alpha oscillation tend to stabilize when vegetation density reaches specific thresholds (12%–24%) on average when exposed to a controlled vegetation density under various indoor settings. We propose that the difference in perceived restoration in our results may be attributed to the distinction between subjective and objective measures. While some environmental variables may influence participants’ perceived restoration, they may not exert a significant impact on the objective EEG measures in our study. Specifically, variations in indoor settings, such as differences in ceiling height and level of enclosure between the sites, are reported to yield divergent outcomes. Among the variations in indoor settings between the two sites, particularly Site C has the presence of a higher ceiling height and more open enclosure, which are generally preferred by people according to Vartanian et al.^49^, and preferences are known to be related to restoration^8,50^. Thus, the higher ceiling height and open enclosure in Site C may have contributed to the increased perceived restoration. Moreover, although there is no specific study directly investigating the relationship between ceiling height and EEG responses, the results of Kim et al.’s^51^ study suggest no significant differences in the effects of ceiling height on ABR.

Our study acknowledges several limitations that highlight the need for further research in important areas. Given that our experiment was conducted in real-world settings, the control of experimental conditions becomes particularly significant. However, due to the inherent limitations of the real-world setting, we need to address some specific constraints.

First, despite our efforts to minimize undesired influences, such as noise level, odors, temperature, and humidity, participants may still have been affected by varying environmental conditions, potentially acting as confounding factors. Notably, thermal conditions, including temperature, air flow, radiation heat, and humidity^52–54^, as well as noise^55,56^, have been shown to impact restoration and cognition. Real-world environments do not allow for a full control over these environmental conditions, unlike controlled laboratory settings. Therefore, it is essential for future studies to investigate how environmental conditions in real-world settings enhance or diminish the observed benefits from the indoor nature by evaluating the mediating role of environmental conditions in the indoor nature effectiveness.

Second, while we made significant efforts to minimize carry-over effect, as described in detail in the experimental procedure section, we acknowledge the possibility of its presence. In order to prevent participant fatigue and ensure a manageable experiment duration, we deliberately avoided incorporating long washout periods between site visits. Consequently, there exists the possibility that the effects of the indoor nature may have been attenuated or enhanced during the transition periods when participants were traveling between sites. It is recommended that future studies be designed with longer washout periods to further mitigate potential carry-over effects on experimental results.

Third, our primary aim in designing the experiment was to evaluate the restorative effects of the nature sites in comparison to no nature site. Therefore, we did not measure the precise magnitude of the restorative effect in each site by using the pre-exposure EEG activities as a baseline and instead used the EEC activities in no nature site as a baseline. However, including control groups by measuring pre- and post-exposure EEG activity would have allowed for a more precise assessment of the effects of each site.

Nonetheless, field experiments offer substantial advantages in terms of capturing realism and bridging the gap between natural and laboratory settings^57,58^. Moreover, previous studies in cognitive psychology and neuroscience have highlighted the limited ecological validity of laboratory experiments, emphasizing the importance of conducting research that explores human behaviors in field experiments^59–61^. In this regard, our study holds significant value as it contributes to understanding human responses in real-world indoor environments, potentially validating previous findings from environmental surrogate studies utilizing images, videos, and virtual realities.

## Conclusions

This study focused on the restorative and cognitive benefits of nature in indoor environments through real-world experiments. They were measured by the four PRS components, backward digit span scores, and DTR, DAR, TBR, and ABR, based on the EEG recordings. Overall, the PRS-11 results demonstrated a positive effect of indoor nature that natural indoor environments have positive effects on restorative quality. Furthermore, DTR, DAR, TBR, and ABR, based on EEG recordings, were used to explain the positive effect of indoor nature on attention and mental workload. These findings may contribute to the knowledge base for understanding the underlying mechanisms between indoor nature and perceived benefits, and guide designers to create restorative and cognitive enhancing spaces in design practice.

## Methods

### Experimental setup

#### Experiment sites

**Figure 2:**
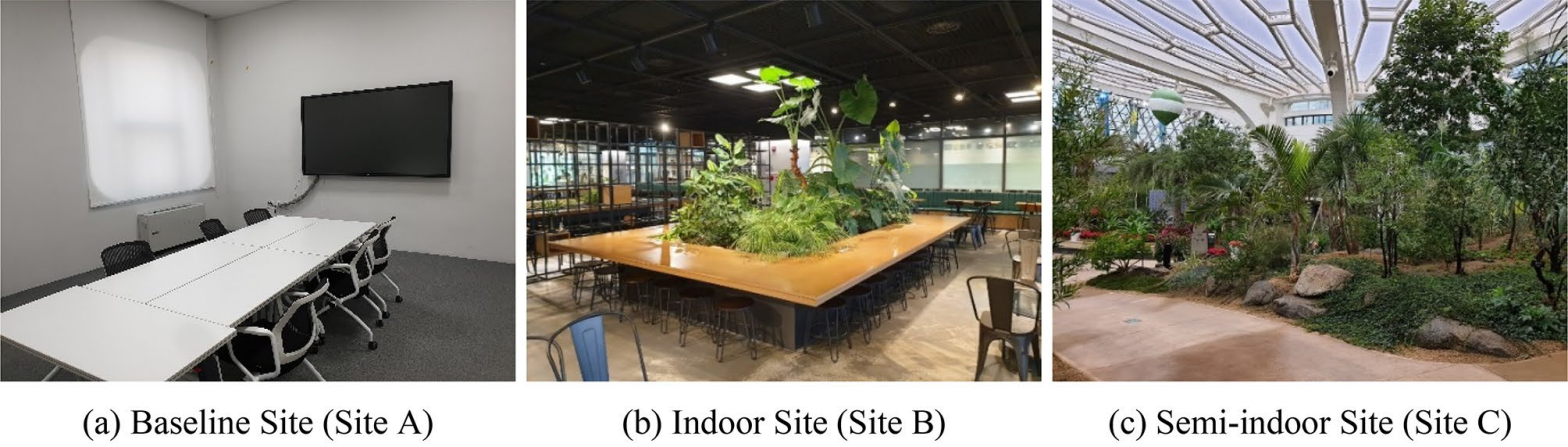
Photos of Experiment Sites. (**a**) Baseline Site (Site A, No Nature); (**b**) Indoor Site (Site B); and (**c**) Semi-indoor Site (Site C).

The experiment sites were at Seoul Botanic Park: a meeting room (baseline site, Site A), a coffee shop (indoor site, Site B), and a greenhouse (semi-indoor site, Site C) (Fig. 2). Site A was an indoor space without any nature, Site B was an indoor space with some vegetation, and Site C was a semi-indoor space with a large amount of vegetation and view of the sky. All three sites were selected based on (1) naturalness, (2) availability during study visits, (3) minimum disturbance of visitors and staff, (4) minimum disturbance of noise, (5) a sufficient level of safety, and (6) proximity: locations within a ten-minute walk.

During the experiment, the attributes influencing the participants’ in situ viewing experience were quasi-controlled. First, we experimented early in the morning to control the exposure of visitors and staff. In addition, we recorded the timeframe of the abrupt appearance of people during the experiment and later removed them from the EEG recordings. Second, we controlled the acoustic stimuli; Site A was 34.92 ± 0.74 dB, Site B was 53.46 ± 1.66 dB, and Site C was 65.86 ± 0.55 dB. To minimize the effects of different noise levels, participants wore earplugs while viewing; however, they removed the earplugs during the backward digit span task to hear the researcher’s voice. Lastly, there were no strong smells, and the overall temperature of the sites was approximately 18°C, with a humidity of 40–50%.

#### Participants

Thirty people (22 females and eight males; average age, 30.1 years ranging 21–48 years) participated in and completed the experiment. All participants were healthy, had no prior history of neurological diseases, and had good vision. The participants were visitors to the Seoul Botanic Park and undergraduate university students. For clean EEG data collection, the participants were requested to comply with the following requirements before their study visit: (1) ensure a good night’s sleep, (2) do not take alcohol and recreational drugs for at least 24 h, (3) avoid arduous activity or caffeine for 12 h, and (4) do not take any food or liquid (except water) for one hour. We obtained informed consents before testing, and the Institutional Review Board of the University of Wisconsin-Milwaukee approved the experimental protocol. All experiments were performed in accordance with the relevant guidelines and regulations.

### Measures

#### PRS-11

This study used PRS to measure restorative quality. The PRS was developed to assess participants’ intuition of restoration related to the surrounding environmental settings^62,63^. Among its various versions, we used PRS-11, which is a shorter version of the PRS scale using fascination, airway, coherence, and scope components^64^. Restorative quality was analyzed using the four categories of the PRS-11 and PRS-General (PRS scores without coherence) on an 11-point Likert scale (0 = not at all and 10 = completely).

#### Backward digit span

This study used a backward digit span task to measure cognitive ability. Participants listened to a digit span and repeated it backward from the last digit to the first digit. The first span started with four digits and increased to ten digits until participants failed to repeat the span backward without any error in two successive trials. The backward digit span task score was equal to the maximum number of digits repeated, and a single succeeding digit was counted as a half point.

#### EEG recording

We measured continuous EEG from 14 saline-base electrode sensors located according to the international 10–20 system, using an Emotiv EPOC. EEG signals were recorded at the anterofrontal (AF3, F7, F3, F4, F8, AF4), frontocentral (FC5, FC6), occipital (O1, O2), parietal (P7, P8), and temporal (T7, T8) sites. We asked the participants to relax and sit still during EEG recordings. However, we did not control for their eye movements, and they spoke and responded to the researcher during the backward digit span task.

#### Experimental procedure

When the participants arrived at Seoul Botanic Park, they had a twenty-minute orientation session that included learning the backward digit span task and signing written consent. They were then assigned to sit at one of the three sites for five minutes, and their resting state EEG was recorded while sitting. The duration of exposure to nature on attention restoration in existing experiments is reported to vary from minutes to hours^1^. Surprisingly, one study found that even a short 40-s break involving viewing nature significantly boosted participants' attention^65^. Previous influential studies have also demonstrated that the effects of nature on reducing stress were noticeable after four minutes of exposure^66^, and improved performance on attentional tasks was observed after six minutes of exposure^7^. In a comprehensive meta-analysis investigating the optimal duration of nature-based activities for mental health benefits^67^, the authors suggested that engaging in nature-related activities for a minimum of five minutes mostly yielded positive psychological outcomes. Additionally, extended experiment period made the pilot test participants bored, which could introduce unwanted influences on the EEG results, such as boredom and mind wandering^68–70^. Therefore, we chose to set our exposure duration to five minutes.

**Figure 3:**
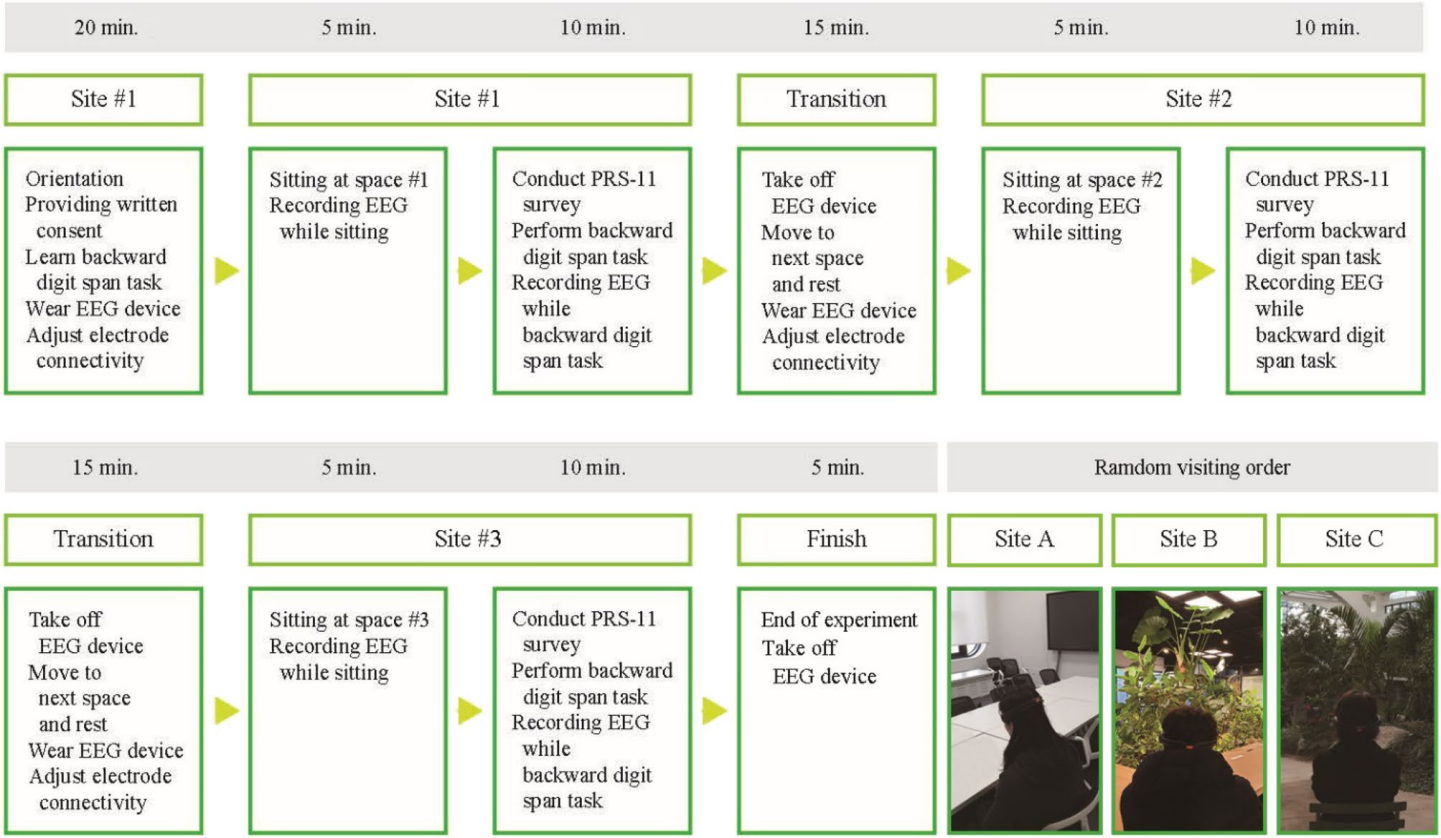
Experimental Procedure.

After five minutes, the participants evaluated PRS-11 and conducted the backward digit span task while recording the EEG. Once the participants finished the experiment, we moved to the next site and rested briefly. The EEG device was then readjusted while resting. Each participant experimented at all three sites, with the order of visits randomized to minimize the carry-over effect. The total duration of the experiment was approximately 100 min, including preparation, sitting, survey, task, move, and break between each site (Fig. 3).

### Data analysis

The effect of indoor nature on restorative quality (PRS-11) and cognitive ability (backward digit span) was quantified by a repeated-measures ANOVA of the three sites. EEG recordings were also analyzed using a repeated-measures ANOVA of the three sites separately for the resting state and cognitive task. All ANOVA results were obtained using SPSS (Version 24).

For EEG analysis, we calculated the anterofrontal EEG responses by averaging the data from AF3, AF4, F3, F4, F7, and F8, and occipital EEG responses by averaging the data from O1 and O2. These calculated EEG responses were then subjected to a fast fourier transformation to calculate the power spectral density (PSD) of the delta (2–4 Hz), theta (4–7 Hz), alpha (8–13 Hz), and beta (13–30 Hz) bands. DTR was calculated by dividing the PSD in delta by that in theta, DAR was calculated by dividing the PSD in delta by that in alpha, TBR was calculated by dividing the PSD in theta by that in beta, and ABR was calculated by dividing the PSD in alpha by that in beta. The data were quantified using EmotivPRO and transferred and pre-processed in MATLAB (Version R2020a) using EEGLAB (Version 2019.1)^71^ and custom scripts.

## Data Availability

The data supporting the findings of this study are included within the article. The datasets used during the study may be made available to qualified investigators upon reasonable request to the corresponding author and with appropriate institutional review board approval.
